# Inference of genetic marker concentrations from field surveys to detect environmental DNA using Bayesian updating

**DOI:** 10.1371/journal.pone.0190603

**Published:** 2018-01-30

**Authors:** Martin T. Schultz

**Affiliations:** Environmental Laboratory, Engineer Research and Development Center, United States Army Corps of Engineers, Vicksburg, Mississippi, United States of America; University of Hyogo, JAPAN

## Abstract

Field studies to detect environmental DNA (eDNA) can be undertaken to infer the presence of a rare or cryptic species in a water body. These studies are implemented by collecting water samples from the water body, processing those samples to isolate genetic material contained in the water sample, and using a laboratory assay to find a species-specific genetic marker within a sample of the genetic material. To date, conventional polymerase chain reaction (PCR) has been one of the most widely used assays in field studies to detect eDNA. This assay is strictly a test for the presence of the genetic marker. It provides no estimate of the concentration of the target genetic marker in the sample or in the environment. Understanding the concentration of a target marker in the environment is a critical first step toward using the results of eDNA field surveys to support inferences about the location and strength of eDNA sources. In this study, the results of eDNA field surveys are combined with a model of the sensitivity of the field survey methods to estimate target marker concentrations using Bayesian updating. The method is demonstrated for Asian carp in the Chicago Area Waterway System (CAWS) using the results of field surveys for eDNA carried out during the period 2009 through 2012, a four-year period during which more than 5,800 two-liter water samples were collected and analyzed using PCR. Concentrations of bighead carp (*Hypophthalmichthys nobilis*) and silver carp (*Hypophthalmichthys molitrix*) eDNA are estimated for twenty hydrologic reaches of the CAWS. This study also assesses the sensitivity of these concentration estimates to evidentiary criteria that limit what evidence is used in Bayesian updating based on requirements for sampling intensity and frequency.

## Introduction

Field surveys to detect environmental DNA (eDNA) specific to a target species can be used to document target species presence [1–4]. For example, the method might be used to identify endangered species habitat without disturbing the organism or to detect aquatic invasive species at low density. Until recently, most eDNA field samples have been analyzed using conventional polymerase chain reaction (PCR) to determine whether or not the target marker is present in the sample. PCR is strictly a test for the presence of the target marker and results of field surveys are reported in terms of the number of water samples testing positive for each target marker. In the absence of information about the amount and distribution of target marker in the environment, it is difficult to make inferences about the location and distribution of potential eDNA sources. Such inferences are a prerequisite for making informed environmental management decisions based on the results of eDNA field surveys.

The aim of this study is to improve how the results of eDNA surveys are interpreted by developing a method of inferring target marker concentrations from conventional PCR results. Recently, developments in PCR technology have enabled researchers to estimate the amount of eDNA in a sample. These studies use quantitative PCR or droplet digital PCR to estimate the number of markers in an aliquot of sample. Some researchers have used these results to back-calculate target marker concentrations in the environmental sample or the environment [5–10]. However, these studies differ in the extent and manner in which they account for losses of eDNA associated with sample processing, including capture, storage, and DNA extraction. These differences serve to underscore that much has yet to be learned about how sampling and analysis methods influence capture and detection rates [11–12]. While it is important to continue research into laboratory assays and field methods that will enable researchers to measure eDNA concentrations, the problem of interpreting surveys completed using conventional PCR remains.

In this study, target marker concentrations are estimated from the results of eDNA field surveys using Bayesian inference, a method of updating knowledge to reflect new evidence. In Bayesian inference, knowledge is represented by a probability distribution that characterizes uncertainty in the value of a parameter. This distribution is updated to a posterior probability distribution each time new evidence is observed. Three pieces of information are required to implement the method. These are a likelihood function, evidence, and a prior probability. The likelihood function used in this study is derived from the outputs of a stochastic model that simulates the sensitivity of the eDNA field survey over a range of potential concentrations. The evidence used to update the prior probability distribution is the fraction of water samples that test positive for the target genetic marker. The prior probability is initially a uniform probability distribution on the ambient concentration of the target marker. Evidence is applied to update the prior distribution to a posterior distribution that characterizes uncertainty in the ambient concentration of the genetic marker. The posterior distribution from the last update becomes the prior distribution for the next update each time new evidence becomes available after a sampling event. The iterative updating procedure yields a refined concentration estimate that incorporates all the evidence observed over the course of sampling events.

The method of estimating concentrations described in this paper was developed during the course of a study to analyze the results of field surveys to detect DNA specific to bighead carp (*Hypophthalmichthys nobilis*) (BHC) and silver carp (*Hypophthalmichthys molitrix*) (SVC) in the Chicago Area Waterway System (CAWS) [13]. BHC and SVC are invasive species of fish that have become well-established in the Illinois River and threaten to invade the Great Lakes, where they could cause economic and ecological damage [14–15]. This paper describes and demonstrates the method, incorporating subsequent refinements in the approach. The analysis is based on more than 5,800 water samples collected between Lake Michigan and Dresden Lock and Dam over the four year period between June, 2009, and October, 2012. During the sampling period, 0.7 percent of water samples tested positive for the BHC target marker and 4.3 percent of water samples tested positive for the SVC target marker. These detection rates provide no insight into how much BHC and SVC eDNA is present in the CAWS or how BHC and SVC eDNA might be distributed in the CAWS. This information is needed to support inferences about the location and strength of eDNA sources, assess whether or not live fish are present in the system, and target BHC and SVC control and eradication efforts in the CAWS.

**Fig 1:**
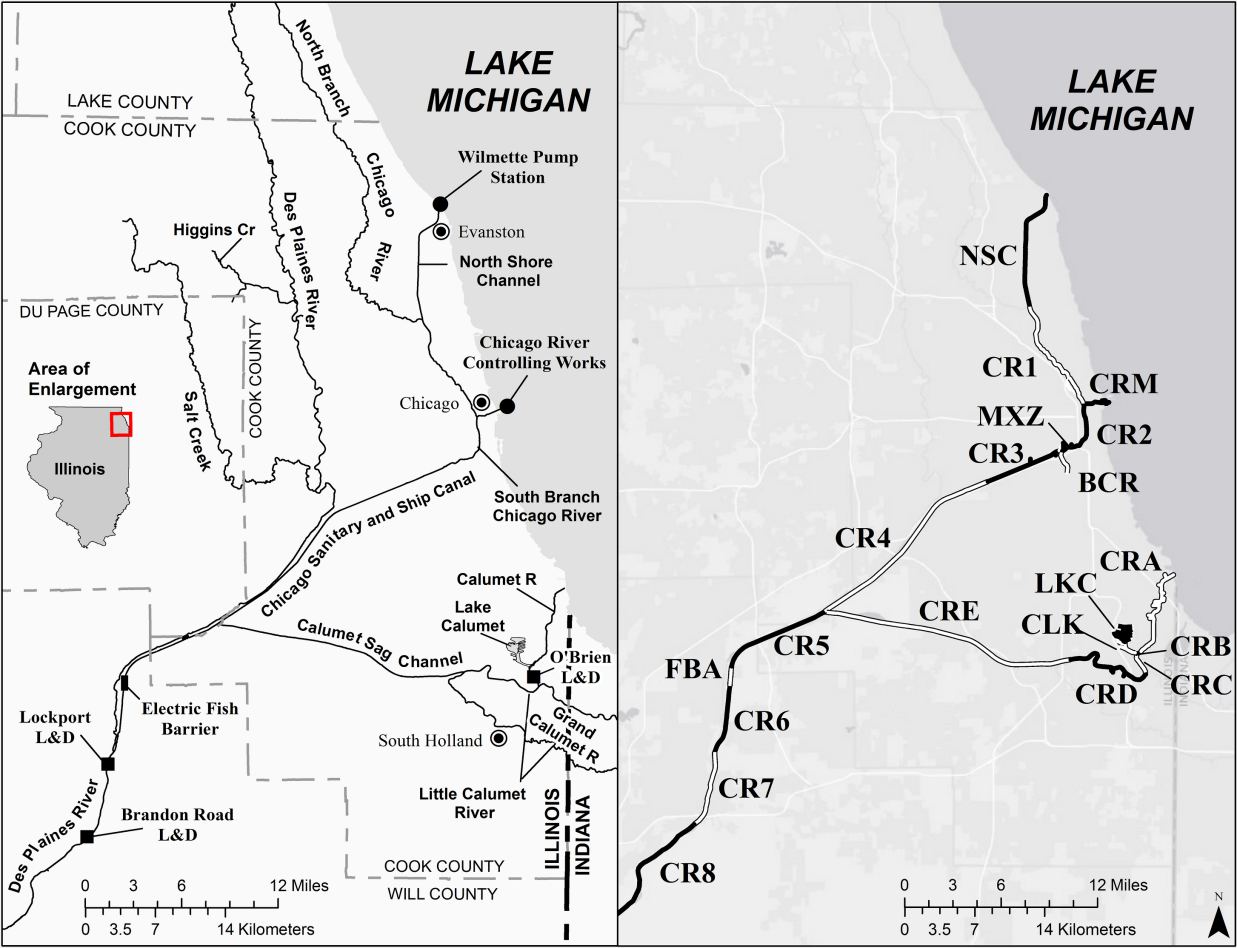
Chicago Area Waterway System (CAWS). The CAWS is divided into twenty main-stem reaches as shown in the right hand panel of the figure.

The CAWS is a navigable network of waterways extending from Lake Michigan to the confluence of the Chicago Sanitary and Ship Canal (CSSC) with the Des Plaines River, south of Lockport Lock and Dam (Fig 1A). Flows from Lake Michigan to the Mississippi River Basin are regulated by three water control structures. These include the Wilmette Pump Station at the head of the North Shore Channel (NSC), the Chicago River Controlling Works in downtown Chicago, and the T.J. O’Brien Lock and Dam on the Little Calumet River. For the purpose of estimating concentrations, the CAWS has been divided into twenty hydrologic reaches, which are distinct segments of the waterway. The location of each reach is illustrated in Fig 1B. Geographic names and verbal descriptions of each reach are provided in Table 1. Reaches are indexed from upstream to downstream following USDA guidelines [16]. Reach boundaries fall at the confluence of two waterway segments, at the outfall of major water treatment plants (e.g., CR3 and CR4), or at lock and dam structures (e.g., CR6, CR7, and CR8). Reach FBA extends the length of an electric fish barrier that is operated by the United States Army Corps of Engineers (USACE) to reduce the probability that BHC and SVC might gain access to Lake Michigan.

**Table 1:** Descriptions of CAWS reaches.

Reach index	Symbol	Description	Surface area (km^2^)	Downstream boundary (decimal degrees)
1	NSC	North Shore Channel from Wilmette Pump Station to its confluence with the North Branch of the Chicago River.	0.35	41.974° N, -87.705° W
2	CR1	North Branch of the Chicago River from its confluence with the North Shore Channel to the South Branch of the Chicago River.	0.67	41.888° N, -87.639° W
3	CRM	Chicago River from Chicago Lock and Controlling Works to just below the Franklin-Orleans Street Highway Bridge.	0.26	41.887° N, -87.637° W
4	CR2	Chicago Sanitary and Ship Canal (CSSC) from the confluence of CRM and CR1 to the turning basin at the base of Bubbly Creek.	0.41	41.844° N, -87.664° W
5	BCR	Bubbly Creek from the turning basin in the main stem of CSSC to its headwaters.	0.09	41.843° N, -87.664° W
6	MXZ	A turning basin at the base of Bubbly Creek that separates BCR, CR2, and CR3.	0.05	41.845° N, -87.666° W
7	CR3	CSSC from Ashland Avenue Bridge to Stickney Water Reclamation Plant (WRP).	0.70	41.845° N, -87.665° W
8	CR4	CSSC from Stickney Water Reclamation Plant to its confluence with the Cal-Sag Channel (CRE).	1.62	41.817° N, -87.753° W
9	CRA	Calumet River from Lake Michigan to the canal linking the Calumet River to Lake Calumet.	1.21	41.665° N, -87.567° W
10	CRB	A turning basin at the confluence of the Calumet River and the Canal to Lake Calumet.	0.15	41.661° N, -87.572° W
11	LKC	Lake Calumet.	1.72	41.673° N, -87.587° W
12	CLK	The navigation canal between Calumet River and Lake Calumet.	0.54	41.663° N, -87.572° W
13	CRC	Little Calumet River south of Turning Basin #5 to its confluence with the Grand Calumet River.	0.26	41.644° N, -87.562° W
14	CRD	Little Calumet River from its confluence with GCR to its confluence with the Cal-Sag Channel.	1.40	41.656° N, -87.652° W
15	CRE	Cal-Sag Channel from the Little Calumet River to the CSSC.	1.88	41.697° N, -87.646° W
16	CR5	CSSC from its confluence with the Cal-Sag Channel to the electric fish barrier.	0.79	41.647° N, -88.059° W
17	FBA	CSSC between the upstream and downstream boundaries of the electric fish barrier (FBA).	0.10	41.629° N, -88.061° W
18	CR6	CSSC from the downstream boundary of the electric fish barrier to Lockport Lock and Dam.	0.53	41.570° N, -88.079° W
19	CR7	CSSC and Des Plaines River from Lockport Lock and Dam to Brandon Road Lock and Dam.	1.08	41.503° N, -88.102° W
20	CR8	Des Plaines River from Brandon Road Lock and Dam to Dresden Lock and Dam.	6.87	41.399° N, -88.281° W

During the four-year monitoring period, the intensity and frequency of sampling varied widely among CAWS reaches (S1 and S2 Tables). When comparing target marker concentration estimates from different reaches, it is difficult to distinguish between estimates that are based on data from well-sampled reaches from estimates that are based on data from poorly-sampled reaches. This issue is addressed through a sensitivity analysis that examines the stability of the target marker concentration estimates to evidentiary criteria that require evidence used in Bayesian updating be based on a minimum number of water samples and minimum number of sampling events. As the criteria are made more stringent, the weakest evidence is dropped from the analysis and fewer iterations of Bayesian updating are used to derive the concentration estimate. If the concentration estimate is sensitive to dropping the weakest evidence, it is not stable. The relative stability of concentration estimates should be considered when comparing concentration estimates from different reaches.

## Materials and methods

Water samples were collected from the CAWS over the course of 68 days during a four-year period from 2009 to 2012. Sample collection and analysis were coordinated by USACE Chicago District. Researchers from the University of Notre Dame collected and analyzed water samples under an agreement with USACE during the period June 2009 through August 2010 [2]. USACE and partner agencies collected and analyzed water samples from August 2010 through 2012. USACE Chicago District has made summaries of the results of these field studies publicly available on its website [17] and provided the data used in this study. The number of water samples and positive detections by CAWS reach and sampling date are reported in S1 and S2 Tables. These summaries of the data were previously described in a report to the Asian Carp Regional Coordinating Committee [13].

The methods used to collect and analyze water samples for the presence of BHC and SVC eDNA are described in a Quality Assurance Project Plan (S1 File). All water samples were collected from public use portions of the CAWS; therefore, no specific permissions were required for collection. No endangered or protected species were involved. Two-liter water samples were collected from just below the water surface and stored on ice in a cooler for processing later in the day. Each sample was filtered through one or more 1.5 μm glass fiber filters, which were then packaged and placed on dry ice for shipment to a laboratory. At the laboratory, eDNA from each water sample was isolated from the filter, purified, and mixed into 100 μl of sterile deionized water. The eluates were stored at minus 20° C for subsequent analysis. Eight aliquots of each eluate were analyzed using PCR. If at least one replicate tested positive for the target marker, amplicons from one positive replicate were sequenced to confirm they matched the BHC or SVC target marker. If so, the water sample was classified as positive for that target marker. Otherwise, the water sample was classified as negative for that target marker.

Estimates of target marker concentration are obtained by Bayesian updating, which is a process for combining prior information about an uncertain parameter, *θ*, with some evidence, *e*, to obtain a posterior probability distribution on that parameter through application of Bayes rule [18]:

$$p(\theta |e)=\frac{p(e|\theta )p(\theta )}{\sum_{\Theta }p(e|\theta )p(\theta )}$$ (1)

The posterior probability of the parameter given the evidence, *p*(*θ*|*e*), is calculated from the prior probability, *p*(*θ*), and the likelihood, *p*(*e*|*θ*), which is the probability of observing the evidence given the parameter. The denominator is the total probability of the evidence, which is the probability of observing the evidence over all possible values of the parameter. The posterior probability estimate updates prior information with evidence obtained through observation. Bayes rule can be applied iteratively to update the parameter estimate each time new evidence is obtained. In iterative applications of Bayes rule, the posterior probability following the last observation becomes the prior probability in the next iteration of updating. Initially, uncertainty in the posterior will be large. Over a sufficient number of monitoring events, uncertainty in the posterior will diminish as the influence of the prior distribution diminishes, and the posterior mean will converge on the unknown mean value of the parameter. This convergence will take longer in systems that exhibit greater variability.

In this application of Bayesian updating, the unknown parameter, *θ*, is the environmental concentration of the target marker and the evidence, *e*, is the fraction of environmental samples testing positive for that genetic marker. The *p*(*θ*) are derived from a prior probability distribution that reflects knowledge about the environmental concentration before new evidence is observed. A uniform distribution, U(*θ*_Min_, *θ*_Max_), is a non-informative prior that represents a lack of information about the concentration. The lower bound of the prior, *θ*_Min_ = 0 copies/L, is a physical minimum concentration. The upper bound is *θ*_Max_ = 3000 copies/L. The rationale for this upper bound is as follows. A model of the sensitivity of the field sampling and analysis protocol, described in [19] indicates that, at concentrations greater than 3000 copies/L, the sensitivity of the protocol used in the CAWS would be equal to one for both BHC and SVC. Therefore, the fraction of water samples testing positive for the target marker would also be one. However, the fraction of water samples that have actually been observed to test positive for each target marker in CAWS eDNA field surveys is generally much less than one. Therefore, it is reasoned that the ambient concentrations of those target markers must be less than this upper bound. Above a concentration of 3000 copies/L, all of the water samples would test positive for the target marker and no inferences about the target marker concentration would be possible because changes in concentration have no influence on the probability of observing a positive assay.

Target marker concentrations are discretized for analysis. Under the uniform distribution, the prior probability of a discretized concentration interval with midpoint *θ* is:

$$p(\theta )=\left(\frac{(\theta +\delta )-\theta_{Max}}{\theta_{Max}-\theta_{Min}}\right)-\left(\frac{(\theta -\delta )-\theta_{Max}}{\theta_{Max}-\theta_{Min}}\right)$$ (2)

The deviation, *δ*, is half the width of the concentration interval. In this application, the concentration is discretized to 601 concentration intervals starting with 0 and with midpoints every 5 copies/L, so *δ* = 2.5 copies/L.

The likelihood is the probability of observing the evidence given the unknown parameter value, which is the environmental concentration of the target marker. A likelihood function is generated using a model that simulates the sensitivity of an eDNA field survey target genetic marker given that it is present in the water body. This model, which was developed in [19], computes sensitivity as a function of a proposed concentration of the target marker in the environment. The model accounts for five steps of sample collection and analysis, including 1) collection of a filtered water sample from the source, 2) extraction and purification of eDNA from the filtered sample, 3) removal of an aliquot from the eDNA elution, 4) fluorescence of PCR amplicons, and 5) sequencing of PCR amplicons to confirm their association with the target species. The model is summarized here, and is parameterized as in [19].

Target marker detection is an event characterized by two possible outcomes, E = {A^+^, A^−^}, where A^+^ is the event for which at least one PCR replicate in the set of PCR replicates tests positive for the target marker and A^−^ is the event for which no PCR replicates test positive for the marker. The probability of each event depends on the concentration of the target marker in the environment, *θ*, and the number of PCR replicates, *K*:

$$p[A^{+}|\theta ]=1-\prod_{k=1}^{K}\left(1-p[R_{k}^{+}|\theta ]\right)$$ (3)

The term *p*[A^+^|*θ*] is the probability that at least one replicate tests positive for the target marker given the environmental concentration. The term $p[R_{k}^{+}|\theta ]$ is the probability that the *k*^th^ replicate tests positive for the target marker given the environmental concentration and is calculated as follows:

$$p[R_{k}^{+}|\theta ]=\sum_{N_{R}}\left[\frac{{\left(\mu_{N_{R}}\right)}^{N_{R}}exp(-\mu_{N_{R}})}{N_{R}!}∙\int_{0}^{N_{R}}\frac{N_{R}^{\left(\alpha_{F}-1\right)}exp(\frac{\alpha_{F}}{\beta_{F}})}{\beta_{F}^{\alpha_{F}}\Gamma (\alpha_{F})}dN_{R}∙\int_{0}^{N_{R}}\frac{N_{R}^{\left(\alpha_{S}-1\right)}exp(\frac{\alpha_{S}}{\beta_{S}})}{\beta_{S}^{\alpha_{S}}\Gamma (\alpha_{S})}dN_{R}\right]$$ (4)

*N*_*R*_ is a random variable describing the initial number of copies of a target marker in a 1 μl PCR replicate drawn from a DNA extract elution with an initial volume, *V*_*E*_ = 100 μl. The first term in the summation on the right-hand side is a Poisson distribution function, which gives the probability that some number of target marker copies, *N*_*R*_, is contained in the PCR replicate. The parameter, $\mu_{N_{R}}$, is the expected initial number of target markers in a PCR replicate and is calculated:

$$\mu_{N_{R}}=\phi ∙N_{S}∙V_{E}^{-1}$$ (5)

The variable *ϕ* characterizes the efficiency of methods used to extract DNA from the water sample, *N*_*S*_ is the uncertain number of copies of the target marker initially captured in the raw water sample, and *V*_*E*_ is the final volume to which the extraction is diluted. *N*_*S*_ is defined by a Poisson distribution:

$$p[N_{s}|\theta ]=\left[{\left(\mu_{N_{S}}\right)}^{N_{S}}∙exp(-\mu_{N_{S}})\right]/N_{S}!$$ (6)

The parameter, $\mu_{N_{S}}$, is the expected number of target marker copies in a water sample from the source and is a function of the environmental concentration of the target genetic marker and the volume of water sampled: $\mu_{N_{S}}=\theta ∙V_{S}$.

The second and third terms in the summation on the right hand side of Eq 4 describe the probability of two events that must occur before a replicate is classified as positive. The first event is fluorescence, the amplicons produced by the PCR reaction must produce visible fluorescence on an agarose gel. The second event is successful sequencing of the amplicons causing fluorescence to confirm their identity. Sequences are compared to representative sequences in GENBANK [20]. When more than one PCR replicate is used, only the amplicons from the PCR replicate producing the largest fluorescent signal are sequenced. The probability of each event is modeled using a gamma density function. The parameters of each gamma density function (α_*F*_, β_*F*_, α_*S*_, and β_*S*_) were estimated from the results of laboratory experiments specifically designed for that purpose [19]. The integrals are solved numerically to compute the cumulative probabilities of visible fluorescence and successful sequencing.

The likelihood function is generated by Monte Carlo simulation of the detection probability model. At each potential target marker concentration, ν = 400,000 realizations of field sampling protocol sensitivity are calculated. These realizations of the sensitivity are then sorted into 101 bins, each representing one potential level of sensitivity between 0 and 1 (e.g., 0.00, 0.01, 0.02,…, 0.99, 1.00). The likelihood of observing some fraction of water samples testing positive at a particular genetic marker concentration is equal to the fraction of realizations in each bin at that concentration. The likelihoods are summarized in a table with 60,701 elements. The table contains one likelihood for each of the 101 potential sensitivity intervals between 0 and 1 at each potential concentration interval between 0 and 3000 copies/L. In principle, it should be possible to observe all levels of sensitivity over the entire range of target marker concentration, except perhaps at the lowest and highest levels of genetic marker concentration.

The value of the likelihood in any particular sensitivity interval should be 0 only if it is impossible to observe that particular level of sensitivity at the given concentration. In practice, however, the calculated likelihood of many sensitivity intervals may be 0 because of sampling error, which arises because there are an insufficient number of Monte Carlo samples to realize all possible outcomes of a random process. The presence of likelihoods equal to 0 at levels of sensitivity and concentration that are in fact possible may lead to bias in concentration estimates. The likelihood is corrected to ensure that levels of sensitivity that are physically possible but that were not realized in the solution have non-zero likelihoods. This is accomplished by substituting the value 1/2ν = 1.25×10^−6^ for the calculated likelihood of 0 at genetic marker concentrations greater than 0, where ν is the number of Monte Carlo samples. The corrected likelihood table is used in each iteration of Bayesian updating. Likelihood tables for BHC and SVC target markers are provided in S2 File.

The evidence used in Bayesian updating is the fraction of water samples testing positive for the target marker. Each time a reach is sampled and new evidence becomes available, the last posterior probability distribution characterizing uncertainty in the target marker concentration in that reach is updated by applying Bayes rule. The next time this reach is sampled and new evidence becomes available, this posterior distribution will serve as the prior distribution. Target marker concentration is treated as a discretized random variable for the purpose of inference and the Bayesian updating procedure yields a set of probability mass functions over 601 discrete concentration intervals. These results can be unwieldy and difficult to present and interpret; therefore, each posterior distribution is summarized by fitting a gamma probability distribution to the probability mass function using the method of moments. The fitted distributions are used to derive a median concentration and credibility interval for the concentration in each reach.

Sampling intensity, the number of water samples collected during a sampling event, and sampling frequency, the number of sampling events during the monitoring period, vary widely throughout the CAWS (S1 and S2 Tables). Sampling intensity is important because a sufficient number of water samples are required to ensure a high probability of detecting the target marker when the target marker is present at low concentrations [19]. Sampling frequency is important because there is a large amount of uncertainty in the initial prior distribution. If the concentration estimates are based on too few iterations of Bayesian updating, the large variance of the initial prior distribution may bias concentration estimates. After several iterations of Bayesian updating, the influence of the initial prior distribution will dissipate and will not bias the posterior concentration estimate.

Evidence that is based on a small number of water samples can be regarded as weaker than evidence that is based on a large number of water samples. However, this information is not reflected in the evidence. Bayesian updating weights all of the evidence used to derive concentration estimates equally and differences in sampling intensity are not reflected in the uncertainty bounds on the concentration estimates. Therefore, it is not readily apparent which target marker concentration estimates are based on evidence from well-sampled reaches and which target marker concentration estimates are based on evidence from poorly sampled reaches. This issue is addressed through sensitivity analysis, which evaluates the extent to which the weakest evidence used in Bayesian updating might be influencing the penultimate concentration estimate.

The extent to which the weakest evidence used in Bayesian updating might be influencing the concentration estimates is assessed by analyzing how sensitive concentration estimates are to the imposition of two evidentiary criteria, one that requires the fraction of water samples testing positive be based on at least some minimum number of water samples and another that this evidence be observed over at least three sampling events. As the stringency of these criteria is increased, evidence that does not satisfy the criteria is dropped from the analysis and concentration estimates are based on fewer iterations of Bayesian updating. If the concentration estimates in a CAWS reach exhibit large amounts of sensitivity to the imposition of evidentiary criteria, they are not stable. The stability of concentration estimates should be considered when comparing concentrations in different CAWS reaches. Instability of a concentration estimate in a reach is an indication that the frequency and intensity of sampling in that reach are not sufficient to support inference of the concentration.

## Results

The primary result of this study are BHC and SVC target marker concentration estimates in twenty CAWS reaches. This presentation of results is organized as follows. A detailed description of concentration estimates in three heavily sampled CAWS reaches is provided to illustrate how the concentration estimates are derived iteratively over many eDNA sampling events. This is followed by a summary of concentration estimates and uncertainty bounds after the last sampling event in each CAWS reach. These estimates reflect all of the evidence accumulated over the four-year eDNA monitoring period. The sensitivity of concentration estimates to the imposition of evidentiary criteria is evaluated to determine the extent to which the weakest evidence might be influencing these concentration estimates. These criteria require that evidence used in Bayesian updating be based on a minimum number of water samples and sampling events.

**Fig 2:**
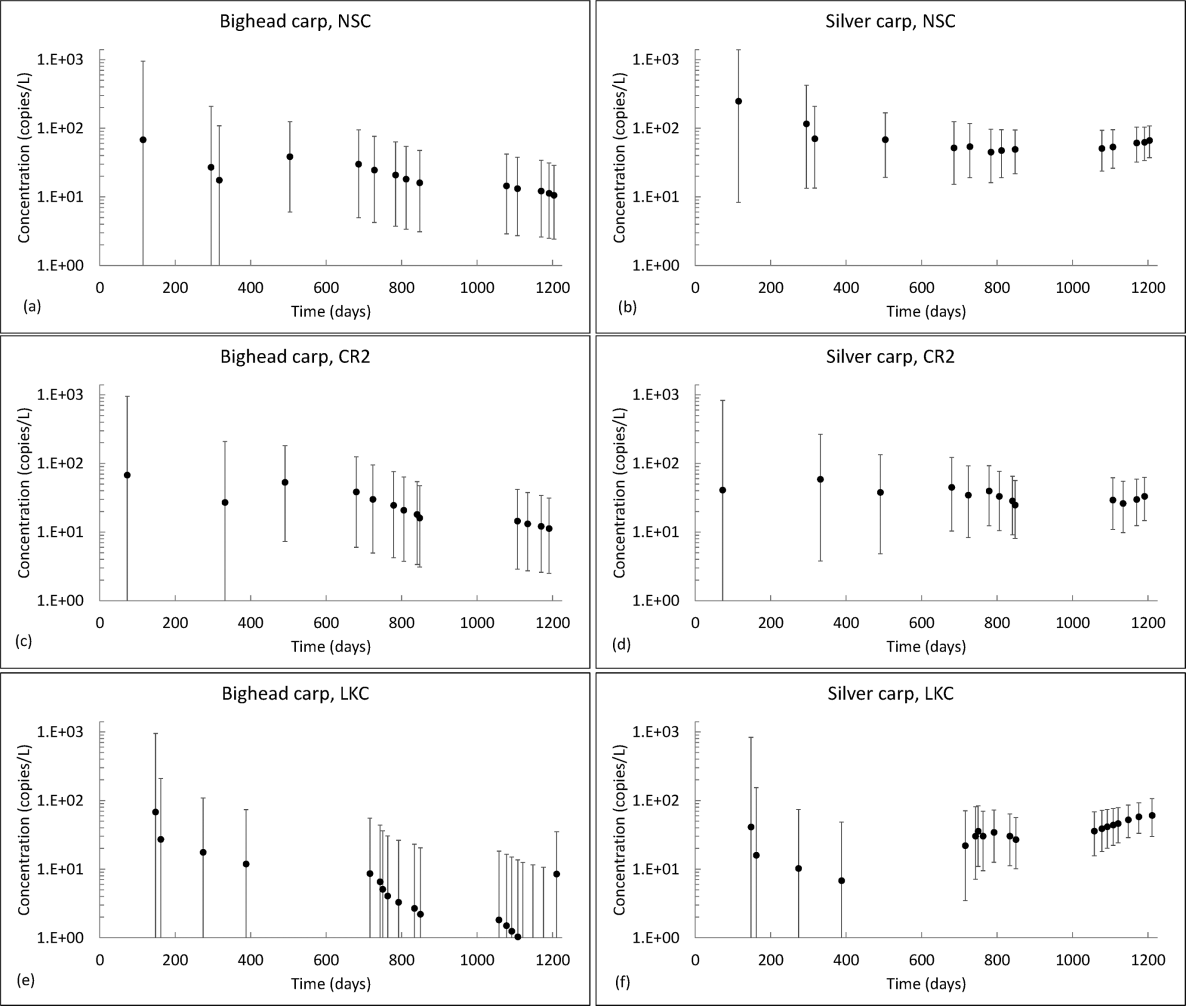
Estimated median BHC and SVC target marker concentration estimates and uncertainty bounds in three CAWS reaches: NSC, CR2, and LKC. The figure shows the medians of the concentration estimates and error bars representing 90-percent credibility intervals on the estimated concentrations. Concentrations are plotted on the number of days since June 29, 2009, the day that the first CAWS water sample was collected.

NSC, CR2, and LKC are among the most heavily sampled reaches in the CAWS. During the period 2009–2012, sixteen sampling events occurred in the NSC, thirteen sampling events occurred in CR2, and nineteen sampling events occurred in LKC. Iterative estimates of BHC and SVC target marker concentrations in these three reaches are summarized in Fig 2. The figure plots the median concentration and 90-percent credibility intervals that were derived following each sampling event on the length of time since the first CAWS water sample was collected (June 29, 2009). The figure shows that BHC target marker concentration estimates are generally lower than SVC target marker concentrations and are associated with greater uncertainty. The figure also shows that the first estimate in each reach tends to be associated with a relatively large amount of uncertainty and this uncertainty diminishes over time as information accumulates over the course of sampling. A more detailed summary of the posterior concentration estimates is provided in supporting information. For these three reaches, S3–S5 Tables list the sampling date, the fraction of water samples testing positive for each target marker, the median BHC and SVC target marker concentrations, the 90-percent credibility interval on concentration estimates, and the parameters of gamma distributions fitted to numerical results.

**Fig 3:**
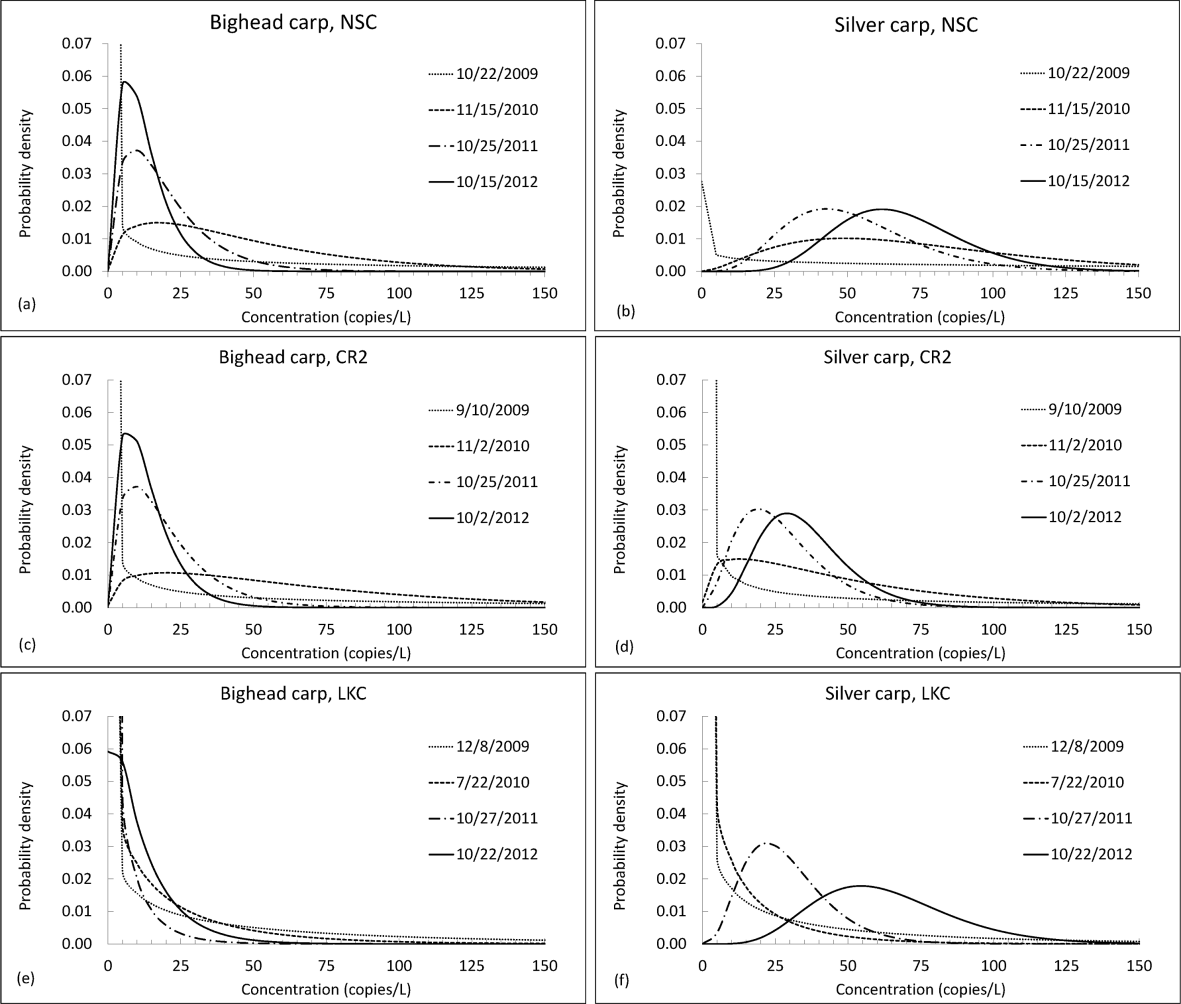
Posterior distributions characterizing BHC and SVC target marker concentrations in three CAWS reaches: NSC, CR2 and LKC. The posterior distributions of target marker concentrations are shown following the last monitoring event each year that samples were collected.

Full posterior gamma distributions characterizing uncertainty in the estimated BHC and SVC target marker concentrations are plotted in Fig 3, showing how the distribution changes as evidence accumulates over time. Distributions for BHC eDNA concentrations exhibit strong positive skew with the highest densities below 25 copies/L because there is very little evidence of BHC eDNA in these reaches. One water sample tested positive for BHC eDNA in LKC on November 15, 2010, one water sample tested positive for BHC eDNA in CR2 on November 2, 2010, and two samples tested positive for BHC eDNA in LKC on October 22, 2012 (S1 Table). In contrast, water samples tested positive for SVC eDNA more frequently (S2 Table), leading to higher concentration estimates. SVC eDNA concentration estimates increase over time because, in each reach, there is a gradual increase in the fraction of water samples testing positive for SVC eDNA during the sampling period.

**Fig 4:**
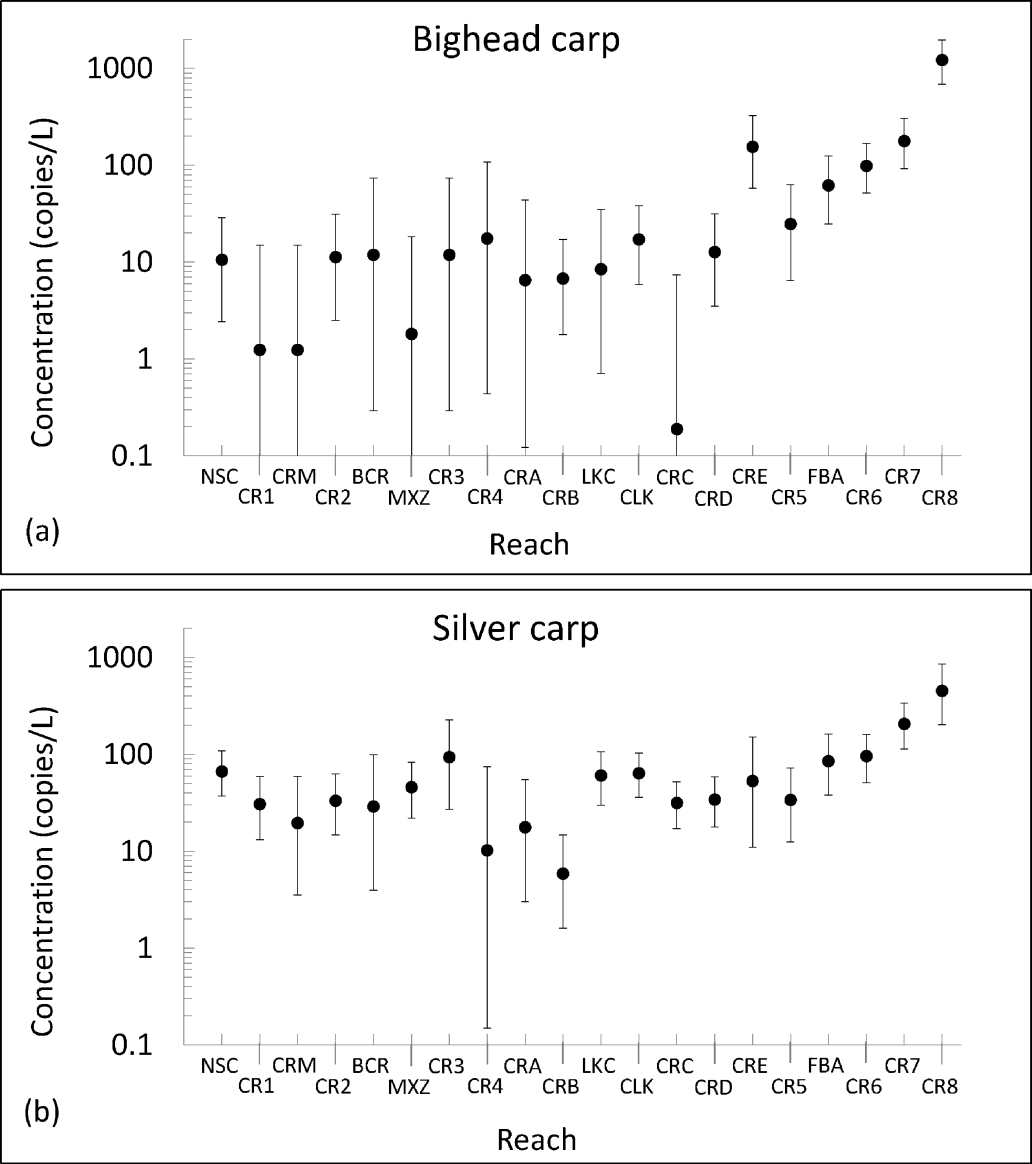
**Posterior target marker concentration estimates following the last sampling event in each CAWS reach for (a) BHC and (b) SVC.** The points represent median concentration estimates and the error bars denote 90-percent credibility intervals.

The median posterior target marker concentration estimate and uncertainty bounds derived following the last sampling event in each CAWS reach are summarized in Table 2 and plotted in Fig 4. Upstream of the fish barrier (to the left of reach FBA), BHC target marker concentrations tend to be lower than SVC target marker concentrations. Uncertainty bounds can span an order of magnitude or more, indicating large uncertainties in these estimates. The upper bounds of target marker concentration estimates are generally less than 100 copies/L. Spatial variability in the concentration can be attributed to the distribution of potential eDNA sources in the CAWS, hydrology, and hydrodynamics. Both species’ target marker concentrations tend to increase in downstream reaches, particularly downstream of the electric fish barrier.

**Table 2:** Posterior target marker concentration estimates following the last sampling event in each CAWS reach.

Reach	BHC Target Marker Concentration (copies/L)		SVC Target Marker Concentration (copies/L)	
	Median	90% Credibility Interval	Median	90% Credibility Interval
NSC	10.6	2.4–28.8	66.5	37.2–108.2
CR1	1.2	0.0–15.0	30.6	13.2–59.2
CRM	1.2	0.0–15.0	19.5	3.5–59.1
CR2	11.3	2.5–31.3	33.1	14.8–62.8
BCR	11.9	0.3–73.7	28.9	4.0–98.5
MXZ	1.8	0.0–18.3	45.9	22.0–82.8
CR3	11.9	0.3–73.7	93.6	27.0–227.1
CR4	17.5	0.4–108.6	10.2	0.1–74.3
CRA	6.5	0.1–43.9	17.7	3.0–54.9
CRB	6.8	1.8–17.2	5.9	1.6–14.7
LKC	8.5	0.7–35.0	60.4	29.9–106.9
CLK	17.2	5.9–38.1	63.7	36.2–102.7
CRC	0.2	0.0–7.4	31.5	17.1–52.2
CRD	12.7	3.5–31.5	34.1	17.8–58.4
CRE	155	58.0–326.2	53.0	11.0–151.2
CR5	24.7	6.4–63.1	33.9	12.4–72.1
FBA	62.0	24.8–125.5	85.3	37.9–161.6
CR6	98.7	51.9–167.9	95.6	51.0–160.7
CR7	178.1	92.2–305.7	205.7	113.1–338.9
CR8	1222.8	690.0–1978.2	451.3	202.5–851.1

The response of median concentration estimates to increasing stringency of the first evidentiary criterion, which requires that evidence used in Bayesian updating be based on a minimum number of water samples, is summarized in Tables 3 and 4. These tables show how the median concentration estimates change as the minimum number of water samples collected from a reach during a sampling event is increased from one to fourteen. For example, imposition of the criterion has no effect on the median concentration in NSC. Estimates in CRM and CR2 exhibit minor sensitivity, but the estimates are stable. CR1 exhibits more sensitivity to the criterion. The response is not monotonic. Median concentration estimates can increase or decrease as the evidentiary criterion is made more stringent. When at least twelve water samples are required, it is no longer possible to estimate target marker concentrations in MXZ because no sampling events consisted of more than twelve water samples. Similarly, when at least fourteen water samples are required, no target marker concentration estimates are possible in BCR and CRB.

**Table 3:** Median BHC target marker concentration (copies/L) estimates following the last sampling event at different levels of the evidentiary criteria. As the criterion that requires a minimum number of water samples becomes more stringent, the median concentration changes because less evidence satisfies the criterion and fewer iterations of Bayesian updating are possible.

Reach	Minimum number of water samples upon which evidence must be based								
	1	2	4	5	6	8	10	12	14
NSC	10.6	10.6	10.6	10.6	10.6	10.6	10.6	10.6	10.6
CR1	1.2	1.2	2.2	6.5	6.5	11.9	27.0	27.0	67.9
CRM	1.2	1.2	1.2	1.2	1.2	1.2	1.2	1.5	1.5
CR2	11.3	11.3	11.3	11.3	11.3	12.1	12.1	12.1	12.1
BCR	11.9	17.5	67.9	67.9	67.9	67.9	67.9	67.9	-^a^
MXZ	1.8	1.8	2.2	2.2	2.7	6.5	27.0	-^a^	-^a^
CR3	11.9	11.9	27.0	27.0	27.0	67.9	67.9	67.9	67.9
CR4	17.5	17.5	17.5	17.5	17.5	17.5	17.5	17.5	17.5
CRA	6.5	6.5	8.6	8.6	8.6	8.6	8.6	11.9	11.9
CRB	6.8	6.8	7.4	8.6	67.9	67.9	67.9	67.9	-^a^
LKC	8.5	8.5	9.7	9.7	9.7	9.7	9.7	9.7	0.7
CLK	17.2	17.2	18.3	18.3	18.3	19.6	19.6	22.8	33.9
CRC	0.2	0.2	0.2	0.2	0.3	0.7	1.5	1.5	5.1
CRD	12.7	12.7	12.7	12.7	12.7	12.7	12.7	12.7	12.7
CRE	155.0	213.9	213.9	213.9	213.9	213.9	213.9	213.9	213.9
CR5	24.7	32.5	32.5	32.5	32.5	32.5	32.5	32.5	32.5
FBA	62.0	62.0	62.0	62.0	71.6	84.7	134.1	84.0	84.0
CR6	98.7	98.7	110.3	110.3	110.3	125.1	144.7	144.7	144.7
CR7	178.1	178.1	255.7	255.7	255.7	321.2	321.2	119.9	119.9
CR8	1222.8	1222.8	1222.8	1222.8	1222.8	1222.8	1222.8	1222.8	1380.6

Shading indicates that these concentration estimates would not be available under an evidentiary criterion requiring at least three iterations of Bayesian updating.

^a^ No concentration could be estimated because no evidence satisfied the criterion for sampling intensity.

**Table 4:** Median SVC target marker concentration (copies/L) estimates following the last sampling event at different levels of the evidentiary criteria. As the criterion that requires a minimum number of water samples becomes more stringent, the median concentration changes because less evidence satisfies the criterion and fewer iterations of Bayesian updating are possible.

Reach	Minimum number of water samples upon which evidence must be based								
	1	2	4	5	6	8	10	12	14
NSC	66.5	66.5	66.5	66.5	66.5	66.5	66.5	66.5	66.5
CR1	30.6	30.6	41.7	3.5	3.5	6.8	15.9	15.9	41.0
CRM	19.5	19.5	19.5	19.5	19.5	19.5	19.5	23.8	23.8
CR2	33.1	33.1	33.1	33.1	33.1	36.5	36.5	36.5	36.5
BCR	28.9	42.2	246.2	246.2	246.2	246.2	246.2	246.2	-^a^
MXZ	45.9	45.9	31.4	31.4	35.3	3.5	15.9	-^a^	-^a^
CR3	93.6	93.6	72.9	72.9	72.9	261.0	261.0	261.0	261.0
CR4	10.2	10.2	10.2	10.2	10.2	10.2	10.2	10.2	10.2
CRA	17.7	17.7	21.8	21.8	21.8	21.8	21.8	28.6	28.6
CRB	5.9	5.9	6.9	4.8	41.0	41.0	41.0	41.0	-^a^
LKC	60.4	60.4	67.7	67.7	67.7	67.7	67.7	67.7	62.2
CLK	63.7	63.7	69.2	69.2	69.2	75.4	75.4	91.1	91.7
CRC	31.5	31.5	31.5	31.5	35.3	46.6	66.0	66.0	47.1
CRD	34.1	34.1	34.1	34.1	34.1	34.1	34.1	34.1	34.1
CRE	53.0	80.7	80.7	80.7	80.7	80.7	80.7	80.7	80.7
CR5	33.9	46.1	46.1	46.1	46.1	46.1	46.1	46.1	46.1
FBA	85.3	85.3	85.3	85.3	102.9	127.5	159.2	181.6	181.6
CR6	95.6	95.6	108.6	108.6	108.6	125.1	125.1	125.1	125.1
CR7	205.7	205.7	245.5	245.5	245.5	245.5	280.3	254.9	254.9
CR8	451.3	451.3	451.3	451.3	451.3	451.3	451.3	451.3	493.4

Shading indicates that these concentration estimates would not be available under an evidentiary criterion requiring at least three iterations of Bayesian updating.

^a^ No concentration could be estimated because no evidence satisfied the criterion for sampling intensity.

The response to the second evidentiary criterion, which requires that there be at least three sampling events to support three iterations of Bayesian updating, is also shown in Tables 3 and 4. As the number of sampling events is increased from one to three, concentration estimates become unavailable in the less-well sampled reaches. Estimates that do not satisfy this criterion are indicated by the shading of cells in Tables 3 and 4. When four or more water samples are required, concentration estimates become unavailable in BCR and MXZ. Similarly, when more than six water samples are required, concentration estimates become unavailable in CRB and when more than ten water samples are required, concentration estimates become unavailable in CR1.

In most CAWS reaches, target marker concentration estimates appear to be insensitive to the imposition of evidentiary criteria (e.g., NSC, CR2). However, in some CAWS reaches, target marker concentration estimates appear to be very sensitive (e.g., CR1, BCR, MXZ, CRB). The relative significance of this sensitivity can be assessed by calculating the ratio of the range in concentration estimates to the minimum concentration estimate. Low values of the sensitivity ratio are desired because they indicate that a reach is sufficiently well-sampled that the criteria have no influence on the estimate. A ratio equal to one indicates that the range is as large as the minimum estimate and a ratio equal to ten indicates that the range is an order of magnitude greater than the minimum estimate. This ratio is reported in Table 5. For BHC target marker concentrations, the ratio is less than or equal to one in 55 percent of CAWS reaches. For SVC target marker concentrations, the ratio is less than or equal to one in 65 percent of CAWS reaches. In general, reaches that exhibit stable BHC target marker concentrations also exhibit stable SVC target marker concentration estimates. Exceptions include LKC and CRC.

**Table 5:** Sensitivity of median BHC and SVC target marker concentration estimates.

Reach	Median BHC target marker concentration (copies/L)				Median SVC target marker concentration (copies/L)			
	Min.^a^	Max.^a^	Range^b^	Ratio^c^	Min.^a^	Max.^a^	Range^b^	Ratio^c^
NSC	10.6	10.6	0.0	0.0	66.5	66.5	0.0	0.0
CR1	1.2	67.9	66.6	55.5	3.5	41.7	38.2	10.9
CRM	1.2	1.5	0.3	0.3	19.5	23.8	4.3	0.2
CR2	11.3	12.1	0.9	0.1	33.1	36.5	3.4	0.1
BCR	11.9	67.9	56.0	4.7	28.9	246.2	217.3	7.5
MXZ	1.8	27.0	25.2	14.0	3.5	45.9	42.4	12.1
CR3	11.9	67.9	56.0	4.7	72.9	261.0	188.2	2.6
CR4	17.5	17.5	0.0	0.0	10.2	10.2	0.0	0.0
CRA	6.5	11.9	5.4	0.8	17.7	28.6	11.0	0.6
CRB	6.8	67.9	61.1	9.0	4.8	41.0	36.3	7.6
LKC	0.7	9.7	9.0	12.9	60.4	67.7	7.4	0.1
CLK	17.2	33.9	16.7	1.0	63.7	91.7	28.0	0.4
CRC	0.2	5.1	4.9	24.5	31.5	66.0	34.5	1.1
CRD	12.7	12.7	0.0	0.0	34.1	34.1	0.0	0.0
CRE	155.0	213.9	58.9	0.4	53.0	80.7	27.7	0.5
CR5	24.7	32.5	7.8	0.3	33.9	46.1	12.2	0.4
FBA	62.0	134.1	72.1	1.2	85.3	181.6	96.4	1.1
CR6	98.7	144.7	46.0	0.5	95.6	125.1	29.6	0.3
CR7	119.9	321.2	201.2	1.7	205.7	280.3	74.6	0.4
CR8	1222.8	1380.6	157.9	0.1	451.3	493.4	42.2	0.1

^a^ The minimum and maximum concentration estimates are from Tables 3 and 4 for BHC and SVC target markers, respectively.

^b^ The range is the difference between maximum and minimum concentration estimates.

^c^ Ratio is the range divided by the minimum concentration estimate. Higher values of the ratio indicate greater sensitivity to the evidentiary criterion.

## Discussion

This paper has presented BHC and SVC target marker concentration estimates in twenty CAWS reaches. These estimates have been derived from the results of eDNA field surveys and a model of eDNA field survey sensitivity using Bayesian updating. The quality of evidence available to derive these estimates varies widely from reach to reach within the CAWS. Some reaches are very well-sampled, with a relatively large number water samples collected during frequent sampling events, and other reaches are poorly-sampled. When assessing the distribution of eDNA in the CAWS, differences in the quality of data available to derive the concentration estimates should be considered. However, it is difficult to distinguish estimates from well-sampled reaches with estimates from poorly-sampled reaches because information about the number of water samples is lost when calculating the fraction of water samples testing positive for each target marker. Therefore, the sensitivity of concentration estimates has been assessed with respect to the imposition of evidentiary criteria that limit what evidence can be used in Bayesian updating. Concentration estimates in poorly-sampled reaches will exhibit greater sensitivity as the weakest evidence is dropped from the analysis.

Bayesian inference requires specification of an initial prior distribution on the parameter that is being estimated. The prior distribution may reflect a synthesis of existing data about the parameter, an objective statement of what is rational to believe about the parameter, or a subjective statement of what the investigator believes about the value of the parameter [21]. The initial prior probability distribution on the target marker concentration that is used in this study is an objective statement of what is rational to believe about the parameter given what is known from a model of eDNA field survey sensitivity [19]. That model suggests that BHC and SVC target marker concentrations must be less than 3000 copies/L because the fraction of water samples testing positive for each target marker is less than one. At concentrations greater than 3000 copies/L, all water samples would test positive for the BHC and SVC target markers and it would not be possible to estimate target marker concentrations. The influence of the initial prior distribution on the concentration estimate has also been minimized by choosing a non-informative prior, which is a uniform probability distribution. After many iterations of Bayesian updating, the shape and variance of the initial prior distribution appear to have little influence on the posterior concentration estimate.

Mean concentration estimates are insensitive to the number and distribution of water samples taken from a reach during any given sampling event. For example, evidence that ten percent of water samples are positive would lead to the same conclusion whether it is based on 100 samples uniformly distributed throughout a reach or ten samples taken from the same location in the reach. Concentration estimates are also insensitive to the order in which a series of evidence is observed. In other words, a history of *n* sampling events over which evidence is observed in a particular sequence will produce the same concentration estimate as a history of *n* sampling events over which the same evidence is observed in a different sequence. However, the amount of uncertainty in a concentration estimate will be influenced by the degree of consistency in the sequence of evidence. Concentration estimates based on a series of observations that are consistent in terms of what fraction of water samples test positive will exhibit less uncertainty than those that are based on a highly variable sequence of evidence.

The penultimate posterior concentration estimate reflects the full body of evidence that accumulates during the monitoring period and the distribution characterizes uncertainty in the eDNA concentration during the sampling events in that monitoring period. For the purpose of this paper, the four-year monitoring period has been treated as a single period for analysis. All of the monitoring data were lumped into a single period for analysis because the sampling effort was very unevenly distributed over time and space. This approach ensures that at least some eDNA monitoring results are available to estimate concentrations in every reach. This seemed like a reasonable approach because, during the four year period, there were few indications of any changes taking place in the system. For example, there were no outages at the electric barrier, there were no sightings of BHC or SVC upstream of the electric barrier, and with the exception of Lake Calumet near the end of the monitoring period, the fraction of water samples testing positive for BHC or SVC eDNA remained relatively constant.

The disadvantage of treating the four-year monitoring period as a single period of analysis is that it is difficult to observe or measure changes in the ambient target marker concentration over time. The analysis culminates in a single posterior concentration estimate in each reach at the end of the period. An analysis of temporal trends in ambient concentrations would require multiple distinct periods of analysis. For example, the four-year monitoring period could be divided into four discrete periods for analysis and the initial prior distribution could be reset at the beginning of each period to obtain a sequence of four independent concentration estimates. This approach was not used in this study because the spatial and temporal distribution of water samples in the CAWS would have been insufficient to support the estimates of target marker concentration in each year and CAWS reach.

While the analysis described in this paper is not designed to measure trends in ambient target marker concentrations over time, changes in ambient concentration over time may manifest themselves as trends in sequential posterior target marker concentration estimates during the single period of analysis. For example, the sequence of posterior estimates in each of the insets of Fig 2 exhibits an apparent trend. There are at least two possible reasons for such trends. In most cases, the two effects are difficult to distinguish and care should be exercised when interpreting these trends in derived concentration estimates.

Trends in sequential posterior concentration estimates during the monitoring period may be caused either by changes in the ambient concentrations over time or by a gradual accumulation of evidence that, by virtue of its consistency over many iterations of updating, converges on a particular concentration. It can be difficult to distinguish between these causes. For example, Fig 2 shows an apparent trend in the BHC concentrations over much of the sampling period in each reach. On its face, this suggests the concentration is decreasing over time. However, this trend in the derived concentration estimate arises because no BHC eDNA is detected in these reaches over many successive sampling events, confirming that, if eDNA is present in these reaches, concentrations are low. The two effects can be distinguished by examining the history of evidence. In this case, there is no coincident trend in the fraction of water samples testing positive for the target marker, which indicates that this trend is caused by an accumulation of evidence. Had a decreasing trend in the fraction of water samples testing positive coincided with the trend in posterior concentration estimates, this would have suggested that at least a portion of that trend could be the result of changes in ambient concentration.

The penultimate concentration estimate does not reflect differences in sampling intensity and frequency among CAWS reaches during the monitoring period. There are several reasons for differences in sampling frequency and intensity. During the latter part of the sampling period, investigators assumed that BHC and SVC would most likely be found in upstream reaches of the CAWS. Therefore, reaches closest to Lake Michigan were sampled more heavily than downstream reaches. Difference in reach surface area may also explain differences in the frequency and intensity of sampling, with larger reaches being sampled more heavily simply because they have larger surface areas. Regardless of the reasons for these differences in sampling intensity Regardless of the reason for these differences in sampling intensity and frequency, they exist. A sensitivity analysis shows that, in poorly-sampled reaches, concentration estimates may be sensitive to the imposition of evidentiary criteria that limit what evidence is used in Bayesian updating. The sensitivity of these concentration estimates should be considered when comparing concentrations in one reach with those in another reach.

## Conclusions

Bayesian inference provides a useful way of combining prior knowledge with the results of eDNA field surveys to estimate and characterize uncertainty in genetic marker concentrations. Field surveys to detect eDNA using PCR provide information on the number of water samples in which the target marker was detected, but not the ambient concentration of the target marker. Estimates of the ambient target marker concentrations are needed to support inferences about the location and strength of potential eDNA sources, assess whether or not live fish are present in the water body, and target BHC and SVC control and eradication efforts. This paper has described and demonstrated how genetic marker concentrations can be estimated from the results of field surveys that document the presence of environmental DNA in water samples. This paper has also demonstrated how the sensitivity of concentration estimates to the frequency and intensity of sampling effort can be evaluated using criteria that limit what evidence is used in Bayesian updating.

## Supporting information

S1 File Quality Assurance Project Plan. The Quality Assurance Project Plan documents the field sample collection and laboratory analysis methods in the CAWS.
(PDF)

S2 File Likelihood tables for BHC and SVC. The likelihood tables contain the probability of detecting BHC and SVC target markers at potential target marker concentrations between 0 and 3000 copies/L.
(XLSX)

S1 Table Distribution of BHC samples in the CAWS. The elements in the table are the ratio of water samples testing positive for BHC eDNA to the total number of water samples analyzed for BHC.
(PDF)

S2 Table Distribution of SVC samples in the CAWS. The elements in the table are the ratio of water samples testing positive for SVC eDNA to the total number of water samples analyzed for SVC.
(PDF)

S3 Table Estimates of BHC and SVC eDNA concentrations in NSC. This table shows the median and 90% credibility intervals for concentration estimates in NSC at each iteration of Bayesian updating.
(PDF)

S4 Table Estimates of BHC and SVC eDNA concentrations in CR2. This table shows the median and 90% credibility intervals for concentration estimates in CR2 at each iteration of Bayesian updating.
(PDF)

S5 Table Estimates of BHC and SVC eDNA concentrations in LKC. This table shows the median and 90% credibility intervals for concentration estimates in LKC at each iteration of Bayesian updating.
(PDF)
