# Supplementary material for: Inference of genetic marker concentrations from field surveys to detect environmental DNA using Bayesian updating
Source: PLoS One. 2018 Jan 30;13(1):e0190603. doi: 10.1371/journal.pone.0190603 (PMC5790220; doi:10.1371/journal.pone.0190603)
Supplement: S2 Table — The elements in the table are the ratio of water samples testing positive for SVC eDNA to the total number of water samples analyzed for SVC. (PDF) [file pone.0190603.s004.pdf]

S2 Table: Distribution of SVC samples in the CAWS. The elements in the table are the ratio of water samples testing positive for SVC eDNA to the total number of water samples analyzed for SVC.

Event	Date	NSC	CR1	CRM	CR2	BCR	MXZ	CR3	CR4	CRA	CRB	LKC	CLK	CRC	CRD	CRE	CR5	FBA	CR6	CR7	CR8	Total samples
**1**	6/29/2009	-	-	-	-	-	-	-	-	-	-	-	-	-	-	-	-	-	-	-	7/16	16
2	7/10/2009	-	-	-	-	-	-	-	-	-	-	-	-	-	-	-	-	-	-	8/25	7/12	37
3	8/3/2009	-	-	-	-	-	-	-	-	-	-	-	-	-	-	-	0/25	0/9	0/40	-	-	74
4	8/19/2009	-	-	-	-	-	-	-	-	-	-	-	-	-	-	-	-	-	4/23	1/8	-	31
5	8/25/2009	-	-	-	-	-	-	-	-	-	-	-	-	-	-	-	-	2/8	0/18	5/29	-	55
6	9/10/2009	-	0/13	0/73	0/7	-	-	-	-	-	-	-	-	-	-	-	-	-	-	-	-	93
7	9/23/2009	-	-	-	-	-	-	-	-	0/14	-	-	-	0/27	1/44	-	-	-	-	-	-	85
8	10/1/2009	-	-	-	-	-	-	1/3	0/38	-	-	-	-	-	-	0/21	1/28	-	-	-	-	90
9	10/15/2009	-	-	-	-	-	-	-	-	-	-	-	-	-	-	-	-	1/11	0/7	-	-	18
10	10/22/2009	5/45	0/7	-	-	-	-	-	-	-	-	-	-	-	-	-	-	-	-	-	-	52
11	10/29/2009	-	-	-	-	-	-	-	-	-	-	-	-	-	-	-	-	-	0/2	0/3	-	5
12	11/24/2009	-	-	-	-	-	-	-	-	0/11	0/3	0/3	0/3	0/13	0/34	2/40	0/1	-	-	-	-	108
13	12/1/2009	-	-	0/11	-	-	-	-	-	-	-	-	-	-	-	-	-	-	-	-	-	11
14	12/2/2009	-	-	-	-	-	-	-	-	-	-	-	-	-	-	-	-	3/17	6/19	5/10	-	46
15	12/8/2009	-	-	-	-	-	-	-	-	4/46	-	0/14	-	0/5	1/33	-	-	-	-	-	-	98
16	3/30/2010	-	-	-	-	-	-	-	-	0/39	0/2	0/14	-	0/7	1/43	-	-	-	-	-	-	105
17	4/15/2010	-	-	-	-	-	-	-	-	-	-	-	-	0/6	0/61	-	-	-	-	-	-	67
18	4/20/2010	1/67	0/20	-	-	-	-	-	-	-	-	-	-	-	-	-	-	-	-	-	-	87
19	5/12/2010	0/58	-	-	-	-	-	-	-	-	-	-	-	-	-	-	-	-	-	-	-	58
20	5/20/2010	-	-	-	-	-	-	-	-	-	-	-	-	0/9	0/35	-	-	-	-	-	-	44
21	5/27/2010	-	-	1/20	1/45	1/13	1/3	4/34	-	-	-	-	-	-	-	-	-	-	-	-	-	115
22	6/29/2010	-	-	-	-	-	-	-	-	-	-	-	-	-	-	-	-	-	-	10/29	21/31	60
23	7/13/2010	-	-	-	-	-	-	-	-	-	-	-	-	-	-	-	-	4/21	13/22	1/3	-	46
24	7/20/2010	-	-	-	-	-	-	-	-	0/85	0/5	-	-	0/8	-	-	-	-	-	-	-	98
25	7/22/2010	-	-	-	-	-	-	-	-	-	0/3	0/68	0/24	-	-	-	-	-	-	-	-	95
26	10/13/2010	-	-	-	-	-	-	-	-	-	-	-	-	-	-	-	4/54	2/21	6/39	-	-	114

Event	Date	NSC	CR1	CRM	CR2	BCR	MXZ	CR3	CR4	CRA	CRB	LKC	CLK	CRC	CRD	CRE	CR5	FBA	CR6	CR7	CR8	Total samples
27	11/2/2010	-	-	0/27	0/81	0/1	0/5	-	-	-	-	-	-	-	-	-	-	-	-	-	-	114
28	11/8/2010	-	-	-	-	-	-	-	-	-	-	-	-	0/13	1/96	0/1	-	-	-	-	-	110
29	11/15/2010	1/110	0/4	-	-	-	-	-	-	-	-	-	-	-	-	-	-	-	-	-	-	114
30	11/30/2010	-	-	-	-	-	-	-	-	-	-	-	-	-	-	-	1/45	0/6	0/52	-	-	103
31	12/7/2010	-	-	-	-	-	-	-	0/29	-	-	-	-	-	-	-	0/47	-	-	-	-	76
32	5/10/2011	-	-	0/24	1/79	-	0/11	-	-	-	-	-	-	-	-	-	-	-	-	-	-	114
33	5/16/2011	0/111	0/3	-	-	-	-	-	-	-	-	-	-	-	-	-	-	-	-	-	-	114
34	6/15/2011	-	-	-	-	-	-	-	-	-	0/3	4/34	2/12	1/15	0/51	-	-	-	-	-	-	115
35	6/23/2011	-	-	0/24	0/79	0/2	0/9	-	-	-	-	-	-	-	-	-	-	-	-	-	-	114
36	6/27/2011	1/105	0/9	-	-	-	-	-	-	-	-	-	-	-	-	-	-	-	-	-	-	114
37	7/12/2011	-	-	-	-	-	-	-	-	-	0/5	2/32	0/11	0/13	0/41	-	-	-	-	-	-	102
38	7/19/2011	-	-	-	-	-	-	-	-	0/2	0/4	1/32	0/11	0/15	1/50	-	-	-	-	-	-	114
39	8/1/2011	-	-	-	-	-	-	-	-	-	0/4	0/32	0/13	0/8	-	-	-	-	-	-	-	57
40	8/17/2011	-	-	0/23	1/73	0/2	0/9	0/7	-	-	-	-	-	-	-	-	-	-	-	-	-	114
41	8/22/2011	0/108	0/6	-	-	-	-	-	-	-	-	-	-	-	-	-	-	-	-	-	-	114
42	8/30/2011	-	-	-	-	-	-	-	-	-	0/4	1/31	0/14	0/14	0/51	-	-	-	-	-	-	114
43	9/6/2011	-	-	-	-	-	-	-	-	-	-	-	-	-	-	-	0/57	0/5	0/52	-	-	114
44	9/13/2011	-	-	0/24	0/79	-	0/8	-	-	-	-	-	-	-	-	-	-	-	-	-	-	111
45	9/19/2011	2/106	0/8	-	-	-	-	-	-	-	-	-	-	-	-	-	-	-	-	-	-	114
46	10/11/2011	-	-	-	-	-	-	-	-	-	0/13	0/30	0/14	0/5	0/51	-	-	-	-	-	-	113
47	10/18/2011	-	-	1/24	0/79	-	0/11	-	-	-	-	-	-	-	-	-	-	-	-	-	-	114
48	10/25/2011	1/111	0/3	2/19	0/65	-	0/8	0/3	-	-	-	-	-	-	-	-	-	-	-	-	-	209
49	10/26/2011	-	-	-	-	-	-	-	0/124	-	-	-	-	-	-	-	2/104	-	-	-	-	228
50	10/27/2011	-	-	-	-	-	-	-	-	-	0/5	0/31	0/14	0/15	1/51	10/113	0/1	-	-	-	-	230
51	5/22/2012	-	-	-	-	-	-	-	-	-	2/3	5/30	4/16	3/14	3/50	-	-	-	-	-	-	113
52	6/11/2012	1/53	0/4	-	-	-	-	-	-	-	0/5	1/16	2/14	0/7	-	-	-	-	-	-	-	99
53	6/25/2012	-	-	-	-	-	-	-	-	-	0/4	4/30	1/15	2/13	0/52	-	-	-	-	-	-	114
54	7/10/2012	3/55	0/2	0/15	5/35	-	1/7	-	-	-	-	-	-	-	-	-	-	-	-	-	-	114

Event	Date	NSC	CR1	CRM	CR2	BCR	MXZ	CR3	CR4	CRA	CRB	LKC	CLK	CRC	CRD	CRE	CR5	FBA	CR6	CR7	CR8	Total samples
55	7/11/2012	-	-	-	-	-	-	-	-	-	-	2/30	0/6	0/7	-	-	-	-	-	-	-	43
56	7/24/2012	-	-	-	-	-	-	-	-	-	0/4	2/31	1/13	0/8	-	-	-	-	-	-	-	56
57	8/6/2012	-	-	0/15	0/35	-	0/7	-	-	-	-	-	-	-	-	-	-	-	-	-	-	57
58	8/20/2012	-	-	-	-	-	-	-	-	-	0/3	6/32	0/14	2/14	0/51	-	-	-	-	-	-	114
59	9/11/2012	11/53	2/4	12/15	5/35	-	0/7	-	-	-	-	-	-	-	-	-	-	-	-	-	-	114
60	9/17/2012	-	-	-	-	-	-	-	-	-	0/4	8/33	2/13	3/13	3/51	-	-	-	-	-	-	114
61	10/2/2012	7/53	1/4	-	4/31	-	3/7	-	-	-	-	-	-	-	-	-	-	-	-	-	-	95
62	10/15/2012	8/53	0/4	-	-	-	-	-	-	-	-	-	-	-	-	-	-	-	-	-	-	57
63	10/22/2012	-	-	-	-	-	-	-	-	-	3/4	11/12	9/14	6/13	5/42	-	-	-	-	-	-	85
64	6/18/2013	-	-	-	-	-	-	-	-	-	0/4	0/31	6/14	0/14	5/31	-	-	-	-	-	-	94
65	6/19/2013	6/53	0/4	1/15	0/35	-	0/7	-	-	-	-	-	-	-	-	-	-	-	-	-	-	114
66	11/5/2013	-	-	-	-	-	-	-	-	-	0/5	0/32	0/13	1/13	1/51	-	-	-	-	-	-	114
67	11/6/2013	-	-	0/19	-	-	-	-	-	-	-	-	-	-	-	-	-	-	-	-	-	19
68	11/7/2013	1/56	0/1	-	-	-	-	-	-	-	-	-	-	-	-	-	-	-	-	-	-	57
