# Supplementary material for: Inference of genetic marker concentrations from field surveys to detect environmental DNA using Bayesian updating
Source: PLoS One. 2018 Jan 30;13(1):e0190603. doi: 10.1371/journal.pone.0190603 (PMC5790220; doi:10.1371/journal.pone.0190603)
Supplement: S3 Table — This table shows the median and 90% credibility intervals for concentration estimates in NSC at each iteration of Bayesian updating. (PDF) [file pone.0190603.s005.pdf]

S3 Table. Sampling results and concentration estimates for BHC and SVC following each sampling event in NSC. This table shows the median and 90% credibility intervals for concentration estimates in NSC at each iteration of Bayesian updating.

Sampling date	Bighead carp target marker					Silver carp target marker				
	F_{BHC}[a]	Fitted posterior distribution				F_{SVC}[a]	Fitted posterior distribution			
		Median (copies/L)	90% Credibility interval (copies/L)	α[b]	β[b]		Median (copies/L)	90% Credibility interval (copies/L)	α[b]	β[b]
6/29/2009	Prior	1500	150-2850	-	-	Prior	1500	150-2850	-	-
10/22/2009	0.0000	68	0-949	0.4	619.9	0.1111	248	8-1398	0.7	572.8
4/20/2010	0.0000	27	0-209	0.5	103.9	0.0149	116	13-424	1.3	121.4
5/12/2010	0.0000	18	0-109	0.7	47.3	0.0000	70	13-208	1.8	46.8
11/15/2010	0.0091	38	6-125	1.6	31.2	0.0091	68	19-168	2.7	28.6
5/16/2011	0.0000	30	5-95	1.6	23.0	0.0000	52	15-124	2.9	20.5
6/27/2011	0.0000	25	4-76	1.7	18.1	0.0095	54	19-117	3.7	16.1
8/22/2011	0.0000	21	4-63	1.7	14.8	0.0000	45	16-97	3.8	13.0
9/19/2011	0.0000	18	3-54	1.8	12.4	0.0189	47	19-95	4.6	11.1
10/25/2011	0.0000	16	3-47	1.8	10.6	0.0090	49	22-94	5.4	9.7
6/11/2012	0.0000	14	3-42	1.9	9.2	0.0189	51	24-93	6.2	8.6
7/10/2012	0.0000	13	3-38	2.0	8.1	0.0545	53	26-95	6.9	8.1
9/11/2012	0.0000	12	3-34	2.0	7.2	0.2075	61	32-104	8.3	7.6
10/2/2012	0.0000	11	2-31	2.1	6.4	0.1321	62	34-104	9.0	7.2
10/15/2012	0.0000	11	2-29	2.2	5.8	0.1509	67	37-108	9.9	6.9
6/19/2013	0.0000	10	2-27	2.2	5.2	0.1132	68	39-108	10.6	6.6
11/7/2013	0.0000	9	2-25	2.3	4.7	0.0179	67	39-106	11.5	6.0

[a] F_{BHC} and F_{SVC} are the fraction of water samples that test positive for the target genetic marker.
[b] α and β are the parameters of the gamma distribution fitted to numerical results.
