# Supplementary material for: Inference of genetic marker concentrations from field surveys to detect environmental DNA using Bayesian updating
Source: PLoS One. 2018 Jan 30;13(1):e0190603. doi: 10.1371/journal.pone.0190603 (PMC5790220; doi:10.1371/journal.pone.0190603)
Supplement: S4 Table — This table shows the median and 90% credibility intervals for concentration estimates in CR2 at each iteration of Bayesian updating. (PDF) [file pone.0190603.s006.pdf]

S4 Table. Sampling results and concentration estimates for BHC and SVC following each sampling event in CR2. This table shows the median and 90% credibility intervals for concentration estimates in CR2 at each iteration of Bayesian updating.

Sampling date	Bighead carp target marker					Silver carp target marker				
	F_{BHC}[a]	Fitted posterior distribution				F_{SVC}[a]	Fitted posterior distribution			
		Median (copies/L)	90% Credibility interval (copies/L)	α[b]	β[b]		Median (copies/L)	90% Credibility interval (copies/L)	α[b]	β[b]
6/29/2009	Prior	1500	150-2850	-	-	Prior	1500	150-2850	-	-
9/10/2009	0	68	0-949	0.4	619.9	0	41	0-832	0.3	622.1
5/27/2010	0	27	0-209	0.5	103.9	0.022222	59	4-267	0.9	92.8
11/2/2010	0.01234	53	7-181	1.4	48.2	0	38	5-134	1.4	36.7
5/10/2011	0	38	6-125	1.6	31.2	0.012658	45	10-122	2.2	24.5
6/23/2011	0	30	5-95	1.6	23.0	0	35	8-92	2.3	17.8
8/17/2011	0	25	4-76	1.7	18.1	0.013699	40	12-93	3.1	14.5
9/13/2011	0	21	4-63	1.7	14.8	0	33	11-77	3.1	11.8
10/18/2011	0	18	3-54	1.8	12.4	0	28	9-65	3.2	9.9
10/25/2011	0	16	3-47	1.8	10.6	0	25	8-56	3.3	8.4
7/10/2012	0	14	3-42	1.9	9.2	0.142857	29	11-62	4.0	8.0
8/6/2012	0	13	3-38	2.0	8.1	0	26	10-55	4.1	7.0
9/11/2012	0	12	3-34	2.0	7.2	0.142857	30	12-59	4.8	6.7
10/2/2012	0	11	2-31	2.1	6.4	0.129032	33	15-63	5.6	6.3

[a] F_{BHC} and F_{SVC} are the fraction of water samples that test positive for the target genetic marker.
[b] α and β are the parameters of the gamma distribution fitted to numerical results.
