# Supplementary material for: Inference of genetic marker concentrations from field surveys to detect environmental DNA using Bayesian updating
Source: PLoS One. 2018 Jan 30;13(1):e0190603. doi: 10.1371/journal.pone.0190603 (PMC5790220; doi:10.1371/journal.pone.0190603)
Supplement: S5 Table — This table shows the median and 90% credibility intervals for concentration estimates in LKC at each iteration of Bayesian updating. (PDF) [file pone.0190603.s007.pdf]

S5 Table. Sampling results and concentration estimates for BHC and SVC following each sampling event in LKC. This table shows the median and 90% credibility intervals for concentration estimates in LKC at each iteration of Bayesian updating.

Sampling date	Bighead carp target marker					Silver carp target marker				
	F_{BHC}[a]	Fitted posterior distribution				F_{SVC}[a]	Fitted posterior distribution			
		Median (copies/L)	90% Credibility interval (copies/L)	α[b]	β[b]		Median (copies/L)	90% Credibility Interval (copies/L)	α[b]	β[b]
6/29/2009	Prior	1500	150-2850	-	-	Prior	1500	150-2850	-	-
11/24/2009	0	68	0-949	0.4	619.9	0	41	0-832	0.3	622.1
12/8/2009	0	27	0-209	0.5	103.9	0	16	0-154	0.4	85.8
3/30/2010	0	18	0-109	0.7	47.3	0	10	0-74	0.6	35.7
7/22/2010	0	12	0-74	0.7	32.2	0	7	0-49	0.6	23.2
6/15/2011	0	9	0-55	0.6	24.7	0.117647	22	3-71	1.6	17.6
7/12/2011	0	7	0-44	0.6	20.1	0.0625	30	7-81	2.2	15.9
7/19/2011	0	5	0-36	0.6	17.1	0.03125	36	11-84	3.0	13.3
8/1/2011	0	4	0-31	0.5	15.0	0	30	9-70	3.1	10.8
8/30/2011	0	3	0-26	0.5	13.4	0.032258	34	13-73	3.9	9.5
10/11/2011	0	3	0-23	0.5	12.1	0	30	11-63	4.0	8.2
10/27/2011	0	2	0-20	0.5	11.2	0	27	10-56	4.1	7.1
5/22/2012	0	2	0-18	0.4	10.4	0.166667	36	16-68	5.4	7.1
6/11/2012	0	1	0-16	0.4	9.7	0.0625	39	18-72	6.1	6.8
6/25/2012	0	1	0-15	0.4	9.2	0.133333	41	20-74	6.7	6.4
7/11/2012	0	1	0-14	0.4	8.8	0.066667	44	22-76	7.5	6.1
7/24/2012	0	1	0-13	0.3	8.4	0.064516	46	24-78	8.2	5.9
8/20/2012	0	1	0-12	0.3	8.0	0.1875	52	29-86	9.5	5.7
9/17/2012	0	1	0-11	0.3	7.7	0.242424	58	33-92	10.7	5.5
10/22/2012	0.166667	8	1-35	1.1	11.2	0.916667	60	30-107	7.1	9.0

[a] F_{BHC} and F_{SVC} are the fraction of water samples that test positive for the target genetic marker.
[b] α and β are the parameters of the gamma distribution fitted to numerical results.
